# Packages of Care for Depression in Low- and Middle-Income Countries

**DOI:** 10.1371/journal.pmed.1000159

**Published:** 2009-10-06

**Authors:** Vikram Patel, Gregory Simon, Neerja Chowdhary, Sylvia Kaaya, Ricardo Araya

**Affiliations:** 1 London School of Hygiene & Tropical Medicine, London, United Kingdom; 2 Sangath, Alto-Porvorim, Goa, India; 3 Group Health Center for Health Studies, Seattle, Washington, United States of America; 4 Muhimbili University of Health and Allied Sciences, Dar es Salaam, Tanzania; 5 Academic Unit of Psychiatry, University of Bristol, United Kingdom; King's College London, United Kingdom

## Abstract

In the first in a series of six articles on packages of care for mental disorders in low- and middle-income countries, Neerja Chowdary and colleagues discuss the treatment of depression.

*This is the first in a series of articles highlighting the delivery of “packages of care” for mental health disorders in low- and middle-income countries. Packages of care are combinations of treatments aimed at improving the recognition and management of conditions to achieve optimal outcomes.*

## Introduction

Depression is the leading cause of disease burden in most regions of the world [1]. The International Classification of Disease (ICD) 10 diagnostic criteria for depressive episode are shown in Box 1. Somatic presentations are very common, especially tiredness, sleep problems, and aches and pains. Of these, only tiredness is considered a “core” feature in current classifications. Anxiety symptoms often coexist with depressive symptoms, particularly in community or primary care samples. The term “common mental disorders” is used to describe the heterogeneous presentation of anxiety, depressive, and somatic symptoms in these contexts [2].

**Box 1. International Classification of Disease 10 Criteria for Depressive Episode**

F 32: Depressive Episode. In typical mild, moderate, or severe depressive episodes, the patient suffers from lowering of mood, reduction of energy, and decrease in activity. Capacity for enjoyment, interest, and concentration is reduced, and marked tiredness after even minimum effort is common. Sleep is usually disturbed and appetite diminished. Self-esteem and self-confidence are almost always reduced and, even in the mild form, some ideas of guilt or worthlessness are often present. The lowered mood varies little from day to day, is unresponsive to circumstances and may be accompanied by so-called “somatic” symptoms, such as loss of interest and pleasurable feelings, waking in the morning several hours before the usual time, depression worst in the morning, marked psychomotor retardation, agitation, loss of appetite, weight loss, and loss of libido. Depending upon the number and severity of the symptoms, a depressive episode may be specified as mild, moderate or severe. Source: World Health Organization [74].

The World Mental Health Surveys have described the prevalence and help-seeking behaviours of people with depression in a large number of countries [3],[4]. The major observations about the epidemiology of depression from these and other studies on depression can be summarized as follows: (1) the constellation of symptoms used to characterize depression can be identified in all cultures; (2) the prevalence rates of depression vary considerably between populations, with rates ranging from about 6% in China to over 20% in the US; (3) the age of onset is most commonly in young adulthood; (4) the disorder often runs a relapsing or chronic course; (5) the disorder is two to three times more common in women, although a few studies, particularly from Africa, have not shown this female excess; and (6) social factors, particularly related to economic or social disadvantages such as low education and violence, are major determinants of the risk for depression [5].

Depression is the leading neuropsychiatric cause of the burden of disease globally and in low- and middle-income countries (LMIC), and is projected to be, overall, the second leading cause of burden of disease by 2020 [6],[7]. Apart from its profound impact on function, depression is associated with increased mortality (particularly through suicide). The condition is often comorbid with other chronic diseases such as diabetes and is responsible for a significant proportion of the disability associated with these conditions [8]. Depression has been associated with a range of poor health outcomes, including poor infant growth (in the case of maternal depression in some countries in South Asia, for example) and worse physical health (for example, cardiovascular or HIV outcomes through poor adherence) [9],[10].

In this article we focus on the effective management of depression in LMICs, reviewing the evidence on efficacy of treatments and delivery of interventions derived from LMICs to the extent possible. Because that evidence is often limited, we also cite systematic reviews or meta-analyses based on global evidence and key trials from high income countries (HIC) where appropriate. On the basis of our review we propose a package of care—a combination of treatments aimed at improving the recognition and management of conditions to achieve optimal outcomes—for depression.

## The Evidence on the Treatment of Depression

### Detection of Depression

A critical first step in the provision of treatments for any disorder is its effective recognition (Table 1). An extensive literature has examined the validity of depression severity measures in HICs. A systematic review [11] examined the accuracy of several commonly used measures including the Beck Depression Inventory (BDI), the Center for Epidemiologic Studies Depression Scale (CES-D), the Zung Self-Rated Depression Scale (SDS), and the Hopkins Symptom Checklist (HSCL). That review found that all commonly used measures performed well in identifying depression among primary care patients, and no measure was recommended over others. The Patient Health Questionnaire (PHQ) was specifically designed to identify and monitor depression severity among primary care patients. Considerable research in the US and Western Europe supports its validity [12],[13].

**Table 1:** The evidence in support of depression treatment.

Depression Treatment	Evidence from HICs	Evidence from LMICs
Detection and monitoring	Meta-analysis of sensitivity and specificity for nine screening measures including BDI, CES-D, SDS, and HSCL [11]Sensitivity and specificity of two-item PHQ depression screener [12]Meta-analysis of accuracy of nine-item PHQ depression scale [13]	GHQ, K6, and SRQ in primary care in India [14]SRQ for perinatal pression in Ethiopia [15]K6 for postnatal depression in Burkina Faso [16]SRQ for women of childbearing age in Mongolia [22]GHQ in 15-site primary care study [23]HSCL in pregnant women positive for HIV in Tanzania [59]GHQ and SRQ in primary care in Chile and in Brazil [17],[18],[20],[21]CIS-R in the community setting in Chile [19]
Antidepressant pharmacotherapy	Meta-analysis of efficacy for older antidepressants [24]Meta-analysis of efficacy for newer antidepressants [25]	RCT of fluoxetine in Indian primary care patients [75]
Cognitive-behavioural therapy (CBT)	Meta-analyses of efficacy of CBT for depression [39],[40]	RCT of CBT delivered by community health workers for perinatal depression in Pakistan [46]RCT of group CBT for depressed primary care patients in Chile [35]
Interpersonal therapy (IPT)	Systematic review of efficacy of IPT for depression [41]	RCT of group interpersonal psychotherapy [47],[48]
Problem-solving therapy (PST)	Meta-analysis of efficacy of PST for depression [44]	—
Electroconvulsive therapy (ECT)	Meta-analysis of efficacy of ECT for depression [50]	—

Abbreviations: BDI, Beck Depression Inventory; CES-D, Center for Epidemiologic Studies Depression Scale; CIS-R, Revised Clinical Interview Schedule for Depression; GHQ, General Health Questionnaire; HSCL, Hopkins Symptom Checklist; K6, Kessler Distress Scale; PHQ, Patient Health Questionnaire; SDS, Zung Self-Rated Depression Scale; SRQ, Self-Reporting Questionnaire.

A relatively large body of research has examined the validity of depression case-finding measures among primary care patients and community residents, and this body of work has focused on measures with more international lineage including the General Health Questionnaire (GHQ), the Self-Reporting Questionnaire (SRQ), and the Kessler Distress Scale (K6). Studies focused on these measures have been done in LMICs including India [14], Ethiopia [15], Burkina Faso [16], Chile [17]–[19], Brazil [20],[21], Mongolia [22], as well as the 15-site World Health Organization Collaborative study [23], and support the validity of each of these measures in primary care and community settings.

### Drug Therapies

Given requirements for drug licensure in the US and Western Europe, a large research literature examines the efficacy of antidepressants in HICs. In its original guidelines for depression treatment in primary care [24], the US Agency for Healthcare Policy and Research summarized data regarding older antidepressants, with an emphasis on studies conducted in primary care settings. That meta-analysis found strong evidence for the efficacy of antidepressant pharmacotherapy and no evidence of advantage for any specific drug over any other. Two subsequent meta-analyses [25],[26] reached a similar conclusion regarding newer antidepressant drugs.

However, one recent meta-analysis found that differences do exist between various newer antidepressants with sertraline emerging as the drug of choice on the basis of superior efficacy and acceptability and lower cost [27]. Furthermore, there is evidence of the greater tolerability of selective serotonin reuptake inhibitors (SSRIs) over tricyclic antidepressants [28], and, for the treatment of depression in adolescents, fluoxetine has been identified as the antidepressant with the optimal balance of benefit versus harm [29].

Following a therapeutic response, antidepressant continuation treatment for a period of 9 to 12 mo has been found to significantly reduce the risk of relapse/recurrence [30].

Conversely, there are some studies that refute the efficacy of antidepressants in the treatment of depression, attributing the perceived effect to publication bias [31] or to the decreased responsiveness to placebo in severely depressed patients (rather than increased responsiveness to antidepressants) [32]. These findings have led to some investigators recommending the need to enhance transparent reporting of clinical trials and reinvigorate antidepressant drug discovery [33].

The literature supporting the efficacy of antidepressant pharmacotherapies for primary care patients in LMICs is relatively small. A randomized trial in India found fluoxetine superior to placebo (specifically at 2-mo follow up) among primary care patients with raised levels of psychological distress [34]. In two Chilean studies [35],[36],which evaluated stepped care, including antidepressants, for women with depression, significant improvements were seen at 6 mo in the intervention group compared to the control group (70% versus 30% in one study and 34% versus 9% in the other study). Although we are not aware of any evidence that medications proven efficacious in HICs are ineffective in other settings, there is some evidence of interethnic differences in the dosing requirements of antidepressant medication that may be attributable to pharmacogenetic polymorphism [37],[38]. This observation may have important implications for drug side effects and adherence to antidepressant medication, and is an important area of future study.

### Structured Psychotherapies

Several randomized trials from HICs support the efficacy of depression-specific structured psychotherapies. Meta-analyses [39],[40] found strong evidence that cognitive therapy for depression is superior to no treatment or a wait-list control condition and some evidence that it is superior to other unstructured or nonspecific psychotherapies. A systematic review [41] found consistent evidence for the efficacy of interpersonal psychotherapy among depressed outpatients.

Most trials of cognitive therapy and interpersonal psychotherapy have been conducted in specialty mental health clinics. In primary care settings, there is consistent evidence favouring brief psychological treatments over treatment as usual [42]. In addition, behavioural activation has been found to be effective in reducing depressive symptoms and is said to be a relatively simple and potentially feasible form of psychological treatment in nonspecialised health care settings [43]. A meta-analysis [44] found that problem-solving therapy had a strong therapeutic effect, but that this effect varied considerably between studies. Most trials of problem-solving therapy have been conducted in primary care settings. A separate meta-analysis by Cuijpers and colleagues [45] compared all of these structured therapies, finding no evidence that one was superior to any other although they were reported to be superior to nonspecific supportive therapy.

As for the evidence from LMICs, several randomized trials have evaluated the effectiveness of structured psychotherapy programs, mostly comparing adapted psychotherapy programs to usual care or no-treatment control conditions. A randomized trial in Pakistan by Rahman and colleagues showed the effectiveness of a cognitive-behavioural therapy delivered by community health workers [46]. Two trials in Chile [35],[36] found a benefit from stepped-care depression treatment, based upon a structured group cognitive-behavioural therapy program, for women presenting to primary care clinics. Two trials from Uganda showed the effectiveness of group interpersonal psychotherapy for depressed residents of rural villages [47] and among depressed adolescents residing in refugee camps [48]. One primary care-based trial in India found no benefit of problem-solving therapy [34]; poor adherence and the high prevalence of severe psychosocial stressors were said to be possible reasons for this poor response [49].

### Electroconvulsive Therapy

A recent meta-analysis of the efficacy of electroconvulsive therapy (ECT) in depression reported a significant benefit of ECT over antidepressants, placebo, and simulated ECT particularly for severe and resistant forms [50]. This review consisted of evidence from randomized and nonrandomized controlled trials mostly from HICs. Additionally, ECT has been argued to be the treatment of choice for severe depression in late life [51]. However, a prospective naturalistic study found that the rate of remission of depression with ECT in community settings is substantially less than that reported in clinical trials [52]. There are no recent studies of ECT efficacy in LMICs where the practice of ECT may in fact be suboptimal due to poor provider methods such as use of bilateral electrode placement, use of sine wave ECT, lack of EEG monitoring, and in some cases, use of unmodified ECT [53].

## Delivery of Effective Interventions

Depression screening and outcome questionnaires appear to perform well across a wide range of settings. Conventional depression treatments, including medications, structured psychotherapies, and ECT appear to have therapeutic benefits. Although the evidence base for the efficacy of depression treatments is not large in LMICs, the evidence we reviewed suggests that depression treatments are just as effective in more disadvantaged patient populations or under-resourced systems of care. All treatments seem to achieve about the same degree of benefit, with combination treatments (both medication and psychotherapy, and ECT for selected individuals), having greater effect in more severe or monotherapy-resistant cases.

### Interventions to Increase Demand for Services

Stigma and the lack of awareness of mental disorders lead to the under-use of available mental health services (Table 2). In HICs awareness campaigns for depression have sought to address this problem by targeting the public, medical professionals, and educational institutions [54],[55]. In LMICs we found one study that aimed to increase community awareness of mental health problems by implementing a school mental health program [56]. The 4-mo program of mental health education in rural Pakistan was conducted by a team of experts in collaboration with teachers and schoolchildren. The program led to a significant improvement in mental health awareness among students and, through them, other members of the local community. However, the impact of this intervention on increasing demand for services was not reported.

**Table 2:** Delivering depression treatments.

Step	How	By Whom	In What Settings
Increasing patient or consumer demand	Improve mental health literacy and reduce stigma [54]–[56]	Mental health specialists teams [35],[54]–[56],[76]	Schools [56]Public arena [54],[55]General practice, primary care [35],[55]Professional education institutions [54],[55]
Increasing capacity of health care teams	Training of primary care providers/all health care providers (professional and nonprofessional), including support and supervision [57]	Mental health specialists team [35],[46]–[48],[57]	Primary care, GPs, clinics, village health teams [57]
Increasing recognition	Community-based screeningPractice-based screening	Trained interviewers: high school graduates [47]School graduates with 1-wk training [14] GPs	Community [47]Primary care [14]Antenatal clinic [77]
Adapting treatments to increase acceptability or reduce cost	Integration of mental health services with routine health care [57],[61]Provision of culturally acceptable care [64],[78],[79]Group psychotherapy interventions [47],[48]Providing treatment in rural settings [47]Programs for vulnerable populations: children [76] and poor women or mothers [35],[36],[46]Providing treatment in work settings [73]	Community health workers [35],[46]–[48],[64]Locally trained paraprofessionals [76]	Rural community [46]–[48]Primary care [35],[36],[64]Schools: rural and those in violence affected areas [56],[76]
Practice-based programs to deliver efficacious treatments	Collaborative care [62]Stepped care [35],[36]Quality improvement programs [65],[80]Adherence support [66],[81]	Case manager, specialist [62]Nonmedical health worker [35],[36]	Primary care [35],[36],[62],[65],[80]
Community-based programs to deliver efficacious treatments	Group interventions [47],[48],[76]Psychologically informed sessions: cognitive behaviour, person-centred [68],[78]Web/telephone-based psychological treatment [69]	Community health workers [47],[48]Health visitors [82]Peer (mother to mother) support [69]Former service users [69]	Community
Addressing impact of the disorder on other health/social outcomes	Psychosocial stimulation [70],[71]Work-based programs [73],[83]Befriending to prevent suicide [72]	Paraprofessionals [71]Case managers [73]	Community and practice based

### Interventions to Improve Capacity of Health Care Teams

A recent report by the World Health Organisation (WHO) and the World Organisation of Family Doctors (WONCA) on integrating mental health into primary care reviewed training initiatives in different settings [57]. Two examples are described here.

The program in Sembabule, Uganda consisted of specialist outreach services from the hospital to the primary care level providing training and ongoing mentoring of primary care workers to provide mental health services. The primary care workers were trained to identify mental health problems, treat patients with uncomplicated common mental disorders, and provide emergency care and referral services. In-service training of general health workers was found to be especially challenging because of understaffing and hence increased workload resulting from added mental health tasks. This training was complemented by the formation of village health teams consisting of trained volunteers. The formation of these teams turned out to be a key step in the mental health integration process.

A mental health program in Kerala, India faced the added challenge of the transfer of trained health workers from the participating clinics to new locations at regular intervals as mandated by government regulations. Thus, frequent ongoing training of newly arrived health workers led to an added burden on resources. Additionally, for the treatment of a chronic condition such as depression, training alone is ineffective and adequate specialist supervision is a vital component of any training initiative.

### Interventions to Improve the Recognition of Depression

There is evidence that locally validated screening instruments can be used by nonphysician and community health workers. When used in such settings, these instruments have comparable psychometric properties and clinical utility to one another. When systematically adapted for use in the local context, such instruments show good criterion validity against both biomedical diagnostic criteria and local descriptions of depression. One such example is the HSCL. When compared with a locally described syndrome approximating depression derived from ethnographic research, this instrument had a sensitivity of 95% [58], Further, HSCL-25 and its depression subscale the HSCL-15, both had sensitivities of 89% for detection of Diagnostic and Statistical Manual of Mental Disorders, 4th edition (DSM-IV) major depression with specificities of 80% and 79%, respectively [59]. A comparison of five screening questionnaires in a primary care setting in India, administered by a school graduate with 1 wk of training, found that they all had comparable case detection psychometric properties [14].

A recent meta-analysis of studies from HICs suggested that screening may not necessarily be the most efficient method for case detection; it reported that intervention effect sizes were larger when patients were referred by general practitioners (GPs) than when referred through systematic screening [42]. A possible explanation may be that those identified by GPs tend to have more severe symptoms than those identified through screening. The Improving Mood-Promoting Access to Collaborative Treatment (IMPACT) study, which enrolled participants by screening and by GP referrals but used the same entry criteria for both, found equal benefit of the collaborative care intervention (involving a collaboration between the primary care team and a supervising psychiatrist) program in the two groups [60]. In any event, although screening questionnaires are a useful tool in primary care, given the variability in GP practice, they need to be supplemented with clinical judgment.

### Interventions to Increase the Acceptability and Reduce the Costs of Treatments

According to the WHO/WONCA report [57], psychological treatments such as interpersonal therapy and cognitive-behavioural therapy have almost all been developed in HICs and need to be adapted to be culturally relevant in LMICs. For example, the Thinking Healthy Program, an adaptation of cognitive-behavioural therapy, has been successfully adapted and used by Lady Health Workers (women from local communities who are trained to deliver basic health care interventions) in the treatment of postnatal depression in Pakistan [46]. Adaptations were made to render the therapy more acceptable, such as using pictorial information to address low levels of literacy and reducing the training period to address the time pressures for the health workers. Providing psychological treatments in the group setting builds on social mechanisms and networks that are already in place in many nonwestern cultures. For example in Uganda, a group interpersonal therapy program was based on interpersonal triggers for depression and group relationship building, concepts that were judged to be compatible with Ugandan culture, and was adapted for treatment of depression both in adults in a rural community and in adolescent war survivors [47],[48]. Providing these treatments in the rural setting, group setting, and using local community workers to deliver the interventions appear also to be effective methods for reducing costs of treatment [35],[46],[47],[61].

### Practice-Based Interventions to Deliver Efficacious Treatments

Collaborative care models that incorporate systematic identification of patients, active case management by competent staff, and specialist supervision are effective for the integration of depression treatment into primary care [62]. These models have largely been developed in HICs. For example, the IMPACT trial successfully used these principles in the treatment of late life depression in primary care settings [60]. Here, intervention patients had access to a case manager who offered education, care management, antidepressant treatment support, and brief psychotherapy, all done under the supervision of a psychiatrist and a primary care expert. This treatment was significantly more effective than usual care at the end of 12 mo. The model, however, may require alteration in LMIC settings with few or no mental health specialists; in such settings, other types of health practitioners with appropriate training in mental health care may provide leadership for the collaborative care program. A cluster randomized controlled trial of such a collaborative care model is currently being conducted in India [63].

Another practice-based intervention may involve tailoring depression treatment to match individual needs, which was the principle behind the multicomponent stepped care intervention used in primary care management of depression in Chile. Two trials in this setting have produced amongst the highest recovery rates recorded in any depression treatment studies and, notably, did not involve a psychiatrist [35],[36]. The intervention delivered by a nonmedical health worker consisted of psychoeducational groups, treatment adherence support, and pharmacotherapy for those with severe depression. Commentators have noted that stepped-care interventions for depression call for brief psychoeducational interventions as first line treatment, reserving pharmacotherapy for those with more severe or treatment-resistant depression [36],[64].

In addition, practice-based quality improvement programs can help improve delivery of efficacious treatments. These programs have largely been developed in HICs to include strategies such as institutional commitment, training of local leaders and health care staff, and detection of depression. These measures can improve both mental health and quality of care outcomes of primary care depression programs [65]. Finally, a practice-based intervention to improve adherence to medicines is periodic telephone contacts, which was found to reinforce adherence to depression treatment in women attending primary care in Chile [35],[66].

### Community-Based Interventions to Deliver Efficacious Treatments

Evidence for community-based interventions to deliver mental health care derives from both LMICs and HICs. A systematic review of the effects of community-based models of mental health care in LMICs found that these lead to improvement in mental health outcomes and cost saving [67]. The study in rural Pakistan cited earlier showed the effectiveness on maternal depression of cognitive behavioural-based psychological intervention integrated into the routine work of community-based primary level workers [46]. A cluster randomized study conducted in urban and rural areas of the Trent region in the UK showed the effectiveness of training health visitors to detect postnatal depression and deliver psychologically informed sessions in women's homes [68].

Evidence suggests that advances in information and communication technology have increased the range of options available for delivery of interventions in the community. Telephone counselling, for example, can increase accessibility, and is private and nonstigmatising. In a trial in Ontario, Canada, telephone-based support by former service users was found to be effective in women identified as high risk for postnatal depression [69].

### Interventions to Address other Health and Social Outcomes

Other interventions to deliver efficacious mental health care treatments include psychosocial stimulation programs [70],[71] and befriending to reduce suicide [72], which may have a role in mitigating the effects of maternal depression on infant development and of depression on suicidal behaviour. A US trial incorporating depression screening, vigorous outreach, and care management of depressed individuals improved not only clinical outcomes but overall work functioning including job retention [73].

## Packages of Care for Depression

Depression is clearly a global health priority. Improving the recognition of this disorder in clinical populations in LMICs is aided by the successful adaption of depression-screening instruments from HIC settings into settings with few resources and weaker health systems. Our review suggests that evidence-based treatments such as antidepressants and psychotherapy are effective in managing depression; it is important, however, that such treatments are adapted when used in LMICs to increase their acceptability, accessibility, and manage their costs. We propose two packages of care on the basis of the availability of mental health specialist resources (Table 3). The delivery of these treatments should ideally be carried out through an integration of depression programs into existing health services or community settings with task-shifting to nonspecialist health workers to deliver front-line care and a supervisory framework of appropriately skilled mental health workers.

**Table 3:** Packages of care for depression.

Low Resourced Settings	High Resourced Settings
Routine screening for detection	High-risk or routine screening with confirmation of diagnosis by skilled clinician
Psychoeducation	Psychoeducation
Generic antidepressants	Choice of antidepressants
Problem-solving	Choice of brief psychological treatments Electroconvulsive therapy

## Abbreviations

ECT: electroconvulsive therapy
GP: general practitioners
HIC: high income countries
LMIC: low and middle income countries
